# Engineered Stone Fabrication Work Releases Volatile Organic Compounds Classified as Lung Irritants

**DOI:** 10.1093/annweh/wxac068

**Published:** 2022-10-14

**Authors:** Chandnee Ramkissoon, Sharyn Gaskin, Tony Hall, Dino Pisaniello, Graeme Zosky

**Affiliations:** Adelaide Exposure Science and Health, School of Public Health, University of Adelaide, Adelaide, SA 5031, Australia; Adelaide Exposure Science and Health, School of Public Health, University of Adelaide, Adelaide, SA 5031, Australia; Mawson Analytical Spectrometry Services, School of Physical Sciences, University of Adelaide, Adelaide, SA 5000, Australia; Adelaide Exposure Science and Health, School of Public Health, University of Adelaide, Adelaide, SA 5031, Australia; Menzies Institute for Medical Research, College of Health and Medicine, University of Tasmania, Hobart, TAS 7000, Australia

**Keywords:** engineered stone, lung irritants, volatile organic compounds

## Abstract

Engineered stones are often characterized for their crystalline silica content. Their organic composition, particularly that of the emissions generated during fabrication work using hand-held power tools, is relatively unexplored. We forensically screened the emissions from dry-cutting 12 engineered stone products in a test chamber for their organic composition by pyrolysis-gas chromatography-mass spectrometry (GC-MS) plus selected traditional capture and analysis techniques. Phthalic anhydride, which has a Respiratory Sensitization (RSEN) Notation by the American Conference of Governmental Industrial Hygienists (ACGIH), was the most common and abundant compound, at 26–85% of the total organic composition of engineered stone emissions. Benzaldehyde and styrene were also present in all twelve samples. During active cutting, the predominant volatile organic compound (VOC) emitted was styrene, with phthalic anhydride, benzene, ethylbenzene, and toluene also detected. These results have important health implications as styrene and phthalic anhydride are irritants to the respiratory tract. This study suggests a risk of concurrent exposure to high levels of respirable crystalline silica and organic lung irritants during engineered stone fabrication work.

**What’s important about this paper?**

The occupational hazard of exposure to respirable crystalline silica (RCS) during the cutting and finishing of engineered stone has been recently recognized. This study, however, is one of the firsts to assess the release of organic compounds, including volatile organic compounds during the cutting of engineered stone. This study suggests potential concurrent exposure to lung irritants such as styrene and phthalic anhydride and high levels of RCS when actively cutting engineered stone.

## Introduction

The rise in the popularity of engineered stones, also known as artificial or composite stones has been associated with many cases of silicosis and other silica-related diseases among workers exposed to dust containing respirable crystalline silica (RCS) from cutting, grinding, and polishing engineered stone products. Apart from the increased incidence of disease, the high degree of severity of silicosis associated with engineered stonework has been a cause of public health concern. It has been proposed that the high crystalline silica content of engineered stone is not sufficient to explain the severity of disease associated with exposure to these dusts (Leso *et al.*, 2019). These materials also contain pigments and resins, which can make up to 7–25% of their bulk composition (Ramkissoon *et al.*, 2022) and could be co-contributors to disease.

Exposure to multiple, often-concurrent hazards, in the workplace, is a widely recognized phenomenon. In the mining and construction industries, concurrent exposures to noise, and vibration, as well as to airborne hazards such as dust, and chemicals are common (SWA, 2015). The principal hazard identified, so far, for the engineered stone industry is RCS for two reasons: firstly, engineered stones have high crystalline silica content in their bulk material and secondly, the adverse health effects of RCS exposure are well established (SWA, 2020). However, little information is available on the organic nature of the dust emitted from engineered stone fabrication work, which could be contributing to the aetiology of lung disease in this occupational group. There appear to be two published works which have explored the organic composition of bulk engineered stone material, but the speciation of organics released during active machining, which could have health implications, is relatively unexplored (Hall *et al.*, 2021; León-Jiménez *et al.*, 2021).

Accordingly, the aim of our study was to firstly, conduct a preliminary screen of emissions from 12 engineered stone products that were machined using hand-held power tools. Secondly, we aimed to actively sample and screen for 73 volatile organic compounds (VOCs) with an additional targeted analysis for styrene and phthalic anhydride based on their abundance in the first screening stage, and contextualise the health implications of potential exposure.

## Methods

Twelve engineered stone products were dry-cut in a controlled environment as described in Ramkissoon *et al.* (2022). Briefly, each stone slab was clamped safely in an enclosed perspex cabinet (60 × 80 × 80 cm) fitted with glove compartments; a series of 3-mm wide zip-cuts were made into the stone using a hand-held angle grinder fitted with a diamond blade (Metabo 720 W, 10 000 rpm). The respirable dust generated by this process was captured on a pre-weighed 37-mm PVC filter, fitted on a Parallel Particle Impactors (PPI) sampler (No.225–383, SKC Inc., Eighty-Four, PA, USA) with a flow rate of 8.0 L/min.

The characterization of the organic fraction of the respirable dust was done by pyrolytic-gas chromatography-mass spectrometry (PY-GC-MS). 1 mg of sample was thermally desorbed in Microscale Sealed Vessel (MSSV) Pyrolysis tubes at 300°C for 30 min prior to release by cracking the tube within a Quantum MSSV II Pyrolysis injector coupled to an Agilent 5977B/7890 GC-MS. Separation was conducted using a 60m DB5-MS capillary column with helium carrier gas flow at 1 ml/min and a temperature ramp from 35°C to 300°C at 8°C per minute held for 17 min at the maximum. Detection was undertaken in full scan mode from 33 Da to 600 Da at approximately three scans per second with analyte identification using Agilent Chemstation software in conjunction with the NIST 14 Library Database.

The mineralogy and resin contents of the dust emissions were determined by X-ray diffraction (XRD) and thermogravimetric analysis (TGA), respectively, as described in our earlier study (Ramkissoon *et al.*, 2022).

The second part of the study involved actively sampling and screening for VOCs including styrene and phthalic anhydride that was generated during a specific brief fabrication task.

One engineered stone product (ES10) was dry-cut as described above; ES10 was chosen on the basis of its relatively high organic composition. During active cutting, the air was drawn through a charcoal sorbent tube (No. 226-01, SKC Inc., Eighty-Four, PA, USA) attached to an SKC Universal Sample Pump (No. 224-30, SKC Inc., Eighty-Four, PA, USA) running at 0.5 L/min for 30 min. After sampling, the tubes were desorbed with carbon disulphide (CS_2_) and screened for a suite of 73 VOCs (Method WCA.207) by GS-MS at an accredited external laboratory (TestSafe NSW, Australia).

While styrene was identified as part of the 73 VOCs screen, phthalic anhydride (an aromatic carboxylic acid anhydride) was sampled separately during the active cutting of ES10. The UK Health and Safety Executive (HSE) method MDHS62/2 was used. Air was drawn through an IOM inhalable dust sampler (No. 225-70A, SKC Inc., Eighty-Four, PA, USA) fitted with a pre-weighed 25-mm glass-fibre filter (No. 225-702, SKC Inc., Eighty-Four, PA, USA) and Tenax TA sorbent tube (No. 226-35 SKC Inc., Eighty-Four, PA, USA) at 0.5 L/min for 30 min. The filter was subsequently desorbed in acetonitrile (HPLC grade) and the tube was desorbed with acetone and acetonitrile. Analysis was done in house by HPLC-UV with fitted with a Kinetex LC column 5 µm C18 100 Å of 150 × 4.6 mm (Phenomenex Inc., California, USA) (LOD 0.5 µg/ml). Where necessary, laboratory and reagent blanks were included for the analyses.

## Results

The respirable dust generated by dry-cutting twelve engineered stones comprised 8.6–20% resin, and 80–91% RCS content in the form of quartz and cristobalite, as reported in our earlier study (Ramkissoon *et al.*, 2022).

Table 1 shows the most abundant organic compounds that were identified by GC-MS after pyrolysis. Phthalic anhydride was present ubiquitously and most abundantly, varying from 26 to 85% of the total organic composition of the emissions. Benzaldehyde was also observed in all 12 samples (4.0–22%), albeit in lower abundance than phthalic anhydride. Styrene was the third most common organic compound characterized from engineered stone emissions, at 2–6% of the total organic composition. Also detected were propylene glycol, benzoic acid, and its derivatives, acetophenone, and phenol. Squalene was observed in variable amounts of 1.3 and 12% in two samples (Table 1).

**Table 1.** The crystalline silica, resin, and organic compositions of the dust are generated by dry-cutting 12 engineered stones (ES1-ES12). The total respirable crystalline silica (RCS) content was determined by XRD, the resin content by TGA, and the organic composition by pyrolytic GC-MS. Compounds making <1% of the total organic composition are denoted by ‘—’.

Engineered stone	Total RCS	Resin	Organic composition of engineered stone emissions									
			Phthalic anhydride	Styrene	Benzaldehyde	Propylene glycol	Acetophenone	Phenol	Benzoic acid derivatives^a^	HA ester^b^	Squalene	Other^c^
	%	%	%	%	%	%	%	%	%	%		%
ES1	91	9	25.7	4.9	20.3	—	3.1	6.9	34.2	—	—	4.9
ES2	88	12	76.0		4.6	—			—	6.3	—	13.1
ES3	86	14	37.6	4.4	11.3	3.8	1.5	4.0	—	—	—	37.4
ES4	84	16	85.4	2.8	9.0	—			—	—	—	2.8
ES5	90	10	34.6	2.1	6.1	1.9	1.3	2.6	43.4	—	—	8
ES6	87	13	42.6	2.1	8.2	2.3	1.4	1.7	20.5	—	—	21.2
ES7	86	14	64.4	3.5	13.3	—	2.3		1.6	1.3	—	13.6
ES8	91	9	64.1	4.4	10.5	—		3.4	—	—	—	17.6
ES9	88	12	42.6	2.7	4.0	1.1	1.1	1.3	25.3	1.7	—	20.2
ES10	88	12	61.0	5.3	16.9	—	2.8		1.1	—	—	12.9
ES11	84	16	58.6	2.5	10.5	1.9	1.8	2.3	21.2	—	1.3	0.0
ES12	80	20	44.5	2.3	21.6	—	3.6	3.2	—	8.3	12.3	4.2

^a^Derivatives include methyl ester benzoic acid;2-propenyl ester benzoic acid.

^b^Hexanedioic acid, bis(2-ethylhexyl) ester.

^c^Other detectable organics were 1,4-Dioxo-1,2,3,4-tetrahydrophthalazine, 1,1ʹ:3ʹ,1″-Terphenyl, 5ʹ-phenyl, chlorobenzene, and chalcone.

To better understand the species and concentrations of organic compounds generated from active machining (a realistic exposure scenario for workers), VOC emissions were captured during a cutting task of one engineered stone (ES10). ES10 was chosen for its high abundance of phthalic anhydride and styrene in the emissions (Table 1). Based on a 30-min sampling event, a total of 11.2 mg/m^3^ VOCs was captured. Among the 73 quantifiable VOCs, only five were reported above their respective detection limit (WorkCover, 2022). Most notably was styrene (3.57 mg/m^3^), toluene (1.70 mg/m^3^), benzene (1.10 mg/m^3^), and ethylbenzene (0.13 mg/m^3^). Acetone was also detected at a concentration of 4.37 mg/m^3^ (Table 2). Phthalic anhydride (inhalable fraction and vapour), sampled and measured separately to the VOC suite, was detected at 0.32 mg/m^3^. Although the purpose of the study was not to determine a workplace exposure assessment to VOCs, it is noteworthy that, phthalic anhydride exceeded the ACGIH-short-term exposure limit (STEL) of 0.005 mg/m^3^. Phthalic anhydride has also been assigned the notations: Respiratory Sensitization (RSEN), Dermal Sensitization (DSEN), and Skin by the ACGIH (ACGIH, 2022).

**Table 2.** The concentration of volatile organic compounds (VOCs) emitted from the engineered stone ES10 during a brief (30 min) dry-cutting task and corresponding ACGIH (2022) threshold limit values (TLV) for short-term exposure limit (STEL) and 8-hour time weighted-average (TWA) exposure levels.‘—’ denotes no identified TLV.

VOCs detected	ACGIH TLV		
	Concentration	STEL	8-hr TWA
	mg/m^3^	mg/m^3^	mg/m^3^
Phthalic anhydride^a^	0.320	0.005	0.002
Styrene	3.57	85	43
Benzene	1.10	8.0	1.6
Ethylbenzene	0.130	—	87
Toluene	1.70	—	75
Acetone	4.37	88	594

^a^Phthalic anhydride as Inhalable fraction and vapour; has DSEN, RSEN, and SKIN Notations (ACGIH, 2022).

## Discussion

Our study appears to be the first to forensically screen emissions from a range of engineered stones for their organic composition. Phthalic anhydride and styrene were two of the most common compounds, together making up 31–88% of the total organic composition of the emissions by PY-GC-MS. Similar organic species were observed when León-Jiménez *et al.* (2021) and Hall *et al.* (2021) pyrolyzed small pieces of engineered stone samples. Given the role of styrene and phthalic anhydride in the manufacture of polymer and epoxy resins, their presence in the resin-containing engineered stones is perhaps not surprising (WHO, 1999; Tustin *et al.*, 2022). Importantly these organic compounds were also detected during an active-machining task, indicating their potential to volatilize and reach a worker’s breathing zone. Our approach using PY-GC-MS allowed an understanding compositionally of the organic content of the material, while the active-machining task highlighted what could be released under the test conditions using a hand-held tool. It was perhaps understandable therefore to capture lower levels of phthalic anhydride compared to other VOCs during active machining despite its predominance in the pyrolyzed emissions. This might also suggest that phthalic anhydride did not volatilize as easily as the other VOCs, especially at the temperature at which the blade cut through the stone, which Hall *et al.* (2021) measured to be approximately 35–40°C for a similar angle grinder and revolutions per minute (rpm). In fact, Hall *et al.* (2021) suggested that phthalic anhydride might be condensed onto the surface of airborne dust particles. Importantly, our analysis for phthalic anhydride included both particulate and vapour, whereas Hall *et al.* (2021) analysed sorbent tubes only. To our knowledge, results of air sampling for phthalic anhydride during actual engineered stone fabrication work have not been reported. Nonetheless, the detectable presence of styrene and phthalic anhydride in engineered stone emissions has crucial health implications because their occupational exposure has been linked to adverse pulmonary effects, including asthma, bronchiolitis obliterans, decreased lung function as well as sclerosis (Wernfors *et al.*, 1986; Moscato *et al.*, 1987; Nett *et al.*, 2017; Meyer *et al.*, 2018).

It is interesting to note the presence of other hazardous substances emitted during the dry-cutting of an engineered stone slab (Table 2), such as benzene, toluene, and acetone. These were perhaps not unexpected since many are common products of organic decomposition. Benzene, despite being measured at low concentrations, is classified as a Group 1 human carcinogen (IARC, 2018) and exposure to toluene vapour can be detrimental to the central nervous system (Filley *et al.*, 2004). However, for the purpose of this study, we provide further discussion on lung irritants phthalic anhydride and styrene as phthalic anhydride was the most abundant organic compound in the bulk composition and styrene has been reported in safety data sheets and elsewhere as a potential constituent by-product of engineered stone processing (Reed *et al.*, 2019).

Styrene is commonly used in the manufacture of reinforced plastics, synthetic rubber, and certain resins (Collins *et al.*, 2013). Occupational exposure to styrene has been associated with adverse pulmonary health effects such as asthma and bronchiolitis as well as systemic sclerosis in men and women (Boudigaard *et al.*, 2020; Meyer *et al.*, 2018; S. *et al.*, 2020). Also commonly used in the manufacture of resins, phthalic anhydride has a respiratory sensitization notation classifying it as a substance that will induce hypersensitivity of the airways following its inhalation. This could lead to asthma, rhinitis, respiratory irritation, and chronic productive bronchitis (Wernfors *et al.*, 1986). Both styrene and phthalic anhydride have airborne occupational exposure limits set by agencies such as the ACGIH designed to guide worker exposure assessment and ensure that control measures are adequate.

We suggest, for the first time, that concurrent exposure to high levels of RCS and respiratory irritants such as styrene and phthalic anhydride may play a role in the disease burden for engineered stone workers. These organic irritants may be generated as vapours during active machining, as described in this study, and perhaps also during the breakdown of inhaled resin(organics)-containing dust *in situ*. The relative contribution of organic compounds to the aetiology of disease associated with engineered stones should potentially consider both sources of exposure. The mechanisms by which RCS and VOCs interact, if at all, in the human body is currently unknown. Zhang *et al.* (2002) showed that phthalic anhydride can react with functional groups found on biological molecules such as hydroxyl, amine, and thiol groups, giving rise to conjugate formation. The protein-anhydride conjugate may be immunogenic with the anhydride acting as a hapten. The combined inflammatory action of phthalic anhydride, styrene, and reactive crystalline silica may be additive or synergistic, especially if they are at the same surface for interaction with lung cells. In addition, chronic obstructive pulmonary disease (COPD) is a disease associated with engineered stone workers, and irritant-induced COPD is likely to be under-reported (Baur *et al.*, 2012) The effect of co-exposure to RCS and VOCs on human respiratory health is currently an underexplored area which warrants further investigation. These results would have a direct translational impact on occupational hygiene practitioners to recommend solutions to mitigate co-exposures for this occupational group.

## Conclusions

The aim of the study was to forensically examine the organic composition of dust emissions generated by machining 12 engineered stones. Our data showed that active fabrication work on engineered stones generated VOCs such as styrene and phthalic anhydride, which are classified lung irritants. This study, perhaps the first, highlighted the potential for concurrent exposure to high levels of RCS as well as harmful VOCs during engineered stone fabrication work. It may be warranted to concurrently sample for VOCs while undertaking RCS exposure assessments under realistic workplace conditions to better understand the actual exposures workers encounter. Further research could investigate how mixed exposures or co-exposure to VOCs along with RCS influences adverse lung outcomes in this occupational group.

## Data Availability

The data underlying this article are available in the article.
